# An approach to machine learning-based non-invasive hemoglobin estimation using multi-wavelength PPG signal features

**DOI:** 10.3389/fphys.2026.1637455

**Published:** 2026-04-21

**Authors:** Bukao Ni, Chaochao Wang, Yanhong Yang, Xiaojun Ji, Abdulilah Mohammad Mayet, Xiaotian Pan, Jun Sun, Xinjun Miao

**Affiliations:** 1 Department of Critical Care Medicine, Wenzhou Central Hospital, Affiliated to Wenzhou Medical University, Wenzhou, Zhejiang, China; 2 Provincial Key Laboratory of Multimodal Perceiving and Intelligent Systems, Jiaxing University, Jiaxing, China; 3 College of Computer Science and Engineering, Tianjin University of Technology, Tianjin, China; 4 Department of Cardiology, Wenzhou Central Hospital, Affiliated to Wenzhou Medical University, Wenzhou, Zhejiang, China; 5 Electrical Engineering Department, King Khalid University, Abha, Saudi Arabia; 6 Institute of Intelligent Media Computing, Hangzhou Dianzi University, Hangzhou, China; 7 Key Laboratory of Micro-nano Sensing and IoT of Wenzhou, Wenzhou Institute of Hangzhou Dianzi University, Wenzhou, China; 8 Department of Neurosurgery, Wenzhou Central Hospital, Affiliated to Wenzhou Medical University, Wenzhou, Zhejiang, China; 9 Department of Emergency, Wenzhou Central Hospital, Affiliated to Wenzhou Medical University, Wenzhou, Zhejiang, China

**Keywords:** feature extraction, hemoglobin, neural networks, non-invasive measurement, photoplethysmography, red blood cells

## Abstract

**Introduction:**

The determination of hemoglobin (Hb) levels is pivotal in the clinical diagnosis and management of anemia and other blood disorders. Traditionally, Hb measurement requires blood samples, which is invasive and can cause patient discomfort and anxiety. There is a growing demand for non-invasive methods that can reduce patient stress and streamline the monitoring process. This study addresses this need by exploring the use of photoplethysmography (PPG) signals for non-invasive Hb measurement.

**Methods:**

Utilizing raw PPG data from 68 subjects, which include both red and infrared light signals, this research applies feature extraction techniques. Three key features—mean, kurtosis, and skewness—were extracted from each signal type, as applied to the dataset, resulting in a comprehensive dataset of six features per subject. These features, along with demographic data such as gender and age, were used as inputs to a Multilayer Perceptron (MLP) neural network.

**Results:**

The neural network was adept at predicting Hb levels, achieving a Mean Relative Error (MRE) of less than 2.46%.

**Discussion:**

The implications of this research are significant, offering a potential shift in how blood hemoglobin levels are measured in clinical settings. By leveraging feature extraction methods and artificial neural networks, this study not only validates the efficacy of PPG as a non-invasive diagnostic tool but also paves the way for future advancements in medical technology. The successful application of machine learning techniques in this context highlights a pathway towards more patient-friendly, efficient, and accessible health monitoring systems.

## 1 Introduction

Hemoglobin (Hb) concentration is a key biomarker in clinical diagnostics, essential for assessing oxygen-carrying capacity and identifying hematological disorders such as anemia. Conventional Hb measurement techniques rely on invasive blood sampling, which, despite high accuracy, can cause patient discomfort and require laboratory facilities not always available in point-of-care or resource-limited environments. Therefore, developing non-invasive, rapid, and reliable approaches for hemoglobin estimation is of significant clinical importance. Photoplethysmography (PPG), an optical method that detects volumetric changes in blood circulation through light absorption, has emerged as a promising non-invasive alternative. By using red and infrared wavelengths, PPG signals can capture variations in blood composition and flow, indirectly reflecting Hb concentration. The recent use of multi-wavelength PPG (MW-PPG) has further improved the capacity to characterize optical absorption properties associated with different hemoglobin levels, making it a valuable modality for machine learning-based analysis.

Several methodologies for non-invasive Hb estimation have been proposed, broadly classified as imaging-based and PPG-based approaches. Imaging methods typically employ smartphone or camera-based systems to estimate Hb from skin or eye images (Hasan et al., 2018; Fan et al., 2022; Dimauro et al., 2023; Li et al., 2023). For instance, the authors in (Hasan et al., 2018) used artificial neural networks to predict Hb from smartphone videos, while a regression-based smartphone biosensor was developed in (Fan et al., 2022) to determine Hb concentration from RGB color features. Dimauro et al (Dimauro et al., 2023). introduced the Eyes-Defy-Anemia dataset and applied RUSBoost classification for anemia detection, and Li et al (Li et al., 2023). employed hyperspectral imaging to non-invasively quantify Hb, platelets, and bilirubin. Although these imaging-based techniques achieved encouraging results, their accuracy often depends on lighting, exposure, and skin tone, which limit clinical robustness. In contrast, PPG-based approaches are less sensitive to ambient light and more directly capture pulsatile blood flow signals. Various studies have investigated PPG signal analysis using machine learning models to estimate Hb levels (Kavsaoglu et al., 2015; Yuan et al., 2015; Ahsan et al., 2017; Tian et al., 2019; Acharya et al., 2020; Liu et al., 2020; Golap et al., 2021; Hossain et al., 2021; Kumar et al., 2022; Kwon and Kim, 2022; Kesarwani et al., 2023; Zhu et al., 2024). Golap et al (Golap et al., 2021). extracted fingertip PPG signals with a genetic algorithm and applied MGGP for Hb regression. Tian et al (Tian et al., 2019). compared BP-ANN and partial least squares (PLS) methods to improve calibration accuracy. Acharya et al (Acharya et al., 2020). proposed a multi-model stacking regressor and examined the effect of pregnancy status, while Hossain et al (Hossain et al., 2021). developed a two-finger model for glycated Hb estimation with high accuracy. Kwon and Kim (2022) employed gradient boosting and random forest models for Hb prediction, and Liu et al (Liu et al., 2020). designed a portable eight-channel PPG system using BP-ANN and PLS. Similarly, Zhu et al (Zhu et al., 2024). implemented multi-wavelength PPG with AdaBoost and BP-ANN, achieving accurate non-invasive Hb estimation. Other optical and hybrid approaches, such as near-infrared spectrophotometry (Yuan et al., 2015), smartphone-based fingertip imaging (Ahsan et al., 2017), palmar pallor analysis (Kesarwani et al., 2023), and multi-wavelength regression models (Kavsaoglu et al., 2015; Kumar et al., 2022), have contributed to this growing field but still face limitations in precision, cost, or generalizability. Despite these advances, most prior works either rely on expensive optical systems or lack robustness across populations. Hence, there remains a need for a computationally efficient and clinically adaptable model capable of predicting Hb accurately from standard MW-PPG signals.

To address this gap, the present study proposes a machine learning framework based on a Multilayer Perceptron (MLP) neural network, which uses statistical features—mean, kurtosis, and skewness—from red and infrared PPG signals combined with demographic information (age and gender). The objective is to evaluate the model’s ability to estimate Hb levels with high accuracy and low mean relative error, thereby advancing non-invasive diagnostic methods for real-time and patient-friendly hemoglobin monitoring.

The primary hypothesis posits that a Multilayer Perceptron (MLP) neural network, utilizing statistical features (mean, kurtosis, skewness) extracted from multi-wavelength PPG signals, can predict hemoglobin levels with a Mean Relative Error (MRE) below 5%, with the secondary hypothesis that demographic integration (age, gender) further improves performance. Endpoints include MRE (primary), alongside RMSE, R², and error standard deviation for comprehensive evaluation.

The major contribution of this research lies in developing a lightweight Multilayer Perceptron (MLP) model based on an existing publicly available PPG dataset (Abuzairi et al., 2024), achieving a Mean Relative Error (MRE) of 2.55% ± 1.05% SD through subject-level stratified 10-fold cross-validation repeated 100 times independently, despite the modest sample size (n=68 subjects). This preliminary feasibility framework addresses prior limitations in non-invasive hemoglobin estimation by: (1) delivering promising regression accuracy (RMSE ≈ 0.32 g/dL) via targeted dual-wavelength statistical feature extraction (mean, kurtosis, skewness), (2) employing a computationally efficient architecture (~500–1, 000 FLOPs per prediction) suitable for deployment on low-cost edge devices, thereby reducing barriers in resource-constrained clinical or remote settings, and (3) enhancing internal performance stability through demographic augmentation (age and gender), which ablation analysis demonstrates reduces MRE by approximately 14% (from 16.05% to 2.20%). These elements collectively advance non-invasive hemoglobin estimation toward practical, scalable clinical applications, as evidenced by comparisons in subsequent sections.

## 2 Methods

### 2.1 Data description

**Figure 1:**
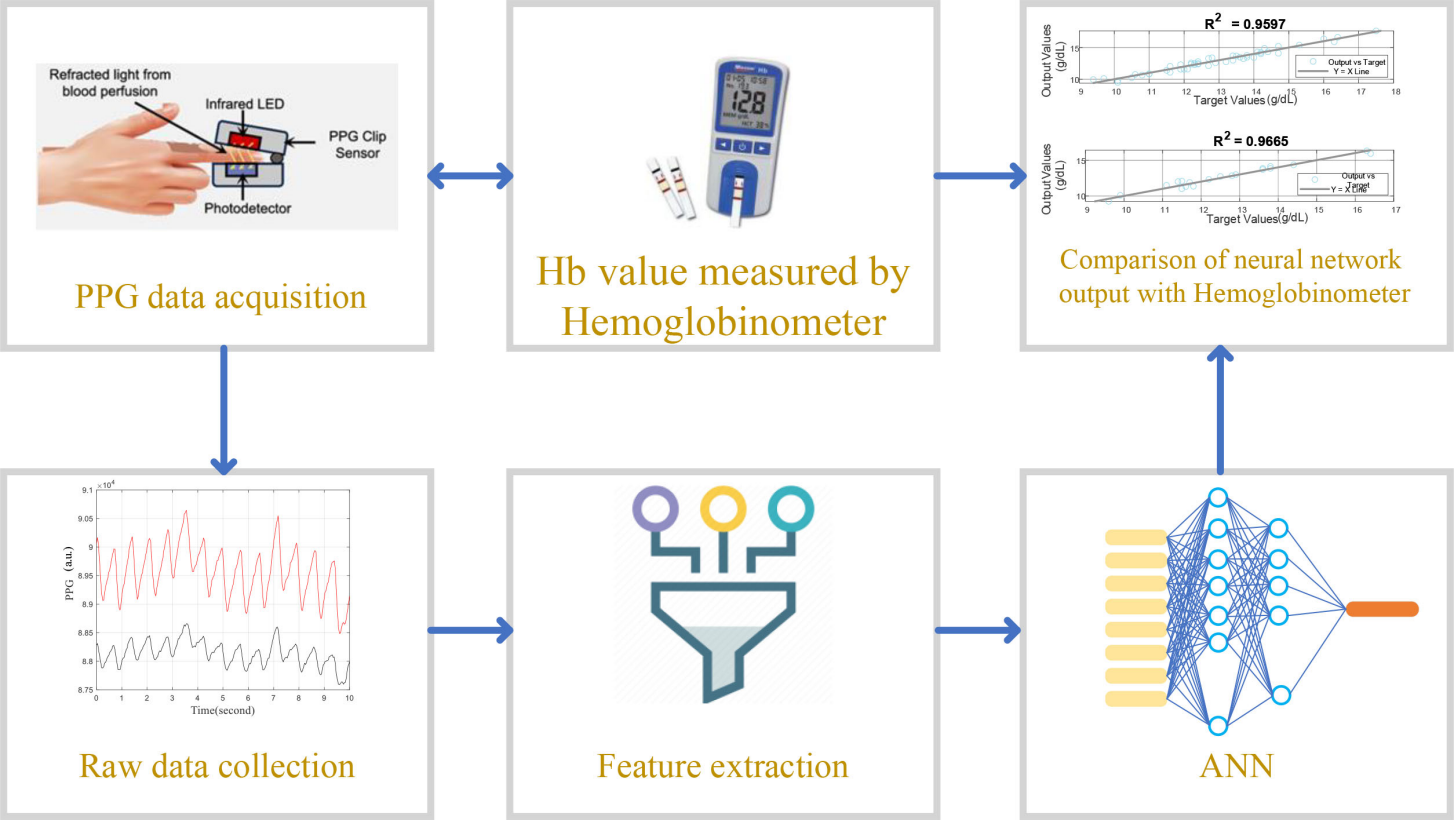
Overview of the non-invasive hemoglobin level prediction process using PPG data, including data acquisition, feature extraction, and comparison of neural network output with hemoglobinometer measurements to evaluate model performance (MRE, RMSE, and R²). Adapted from data processing concepts in Abuzairi et al (Abuzairi et al., 2024), with original visualization including neural network results.

**Figure 1** provides a comprehensive overview of the process used in this study to predict Hb levels non-invasively using PPG data. This diagram outlines the sequential steps involved in the methodology:

1. PPG Data Acquisition: Initially, PPG data is captured using a device that emits light onto the skin to measure blood volume changes. This data is crucial for analyzing blood flow characteristics which relate to hemoglobin levels.
2. Raw Data Collection: The raw signals captured from the PPG device, which include both red and infrared light absorptions, are collected for further analysis.
3. Feature Extraction: From the raw PPG data, key features such as mean, kurtosis, and skewness are extracted. These features help in summarizing the essential characteristics of the PPG signal which are predictive of Hb levels.
4. Hb Value Measurement by Hemoglobinometer: Parallel to the PPG data collection, Hb levels are also measured using a traditional hemoglobinometer. This measurement serves as a benchmark for validating the PPG-based predictions.
5. ANN Implementation: The extracted features along with additional inputs like age and gender are fed into an ANN. The neural network is trained to predict Hb values based on these inputs.
6. Comparison of Neural Network Output with Hemoglobinometer: Finally, the output of the neural network is compared against the standard hemoglobinometer readings to evaluate the accuracy and effectiveness of the PPG-based non-invasive measurement approach.

This figure encapsulates the integration of traditional and modern techniques to enhance the accuracy and non-invasiveness of hemoglobin measurement, illustrating the potential of PPG in clinical diagnostics.

This analysis utilizes a dataset first described by Abuzairi et al. (2024) (Abuzairi et al., 2024), which contains measurements of both PPG and hemoglobin (Hb) levels from 68 subjects recruited at the Primary Health Center Jatiuwung, Tangerang City, Banten, Indonesia. The subjects included a balanced sex ratio of 56% female and 44% male, with ages ranging from 18 to 65 years (distributed across adult age groups, with no pediatric or elderly participants). Health status focused on anemia monitoring, with Hb levels varying from 11 to 17.5 g/dL, representing a mix of normo- and potentially anemic individuals without reported severe comorbidities. The PPG signals were recorded using a device equipped with a MAX30102 sensor module manufactured by Maxim Integrated, enclosed in a 3D-printed fingertip-shaped shell (dimensions: 5 cm × 2.5 cm) to minimize ambient light interference (Abuzairi et al., 2024). The MAX30102 sensor features an 18-bit analog-to-digital converter and low-noise circuitry for ambient light rejection, communicating with an Arduino Uno via the Inter-Integrated Circuit (I2C) protocol (SDA to A4 pin, SCL to A5 pin, power to 3.3V and ground). The dataset comprises 816 records (12 PPG sets per subject, each averaged from 250 raw samples collected at 40 ms intervals over 10 seconds under consistent room temperature and lighting conditions). Hb levels were measured invasively via finger-prick using a Nesco Multicheck 2^®^ hemoglobinometer (Bioptik Technology Inc., Taiwan). These enhancements provide a clearer demographic profile while noting limitations, such as the adult-only age range and focus on mild anemia cases, which may affect generalizability to broader populations.

### 2.2 Dataset collection

**Figure 2:**
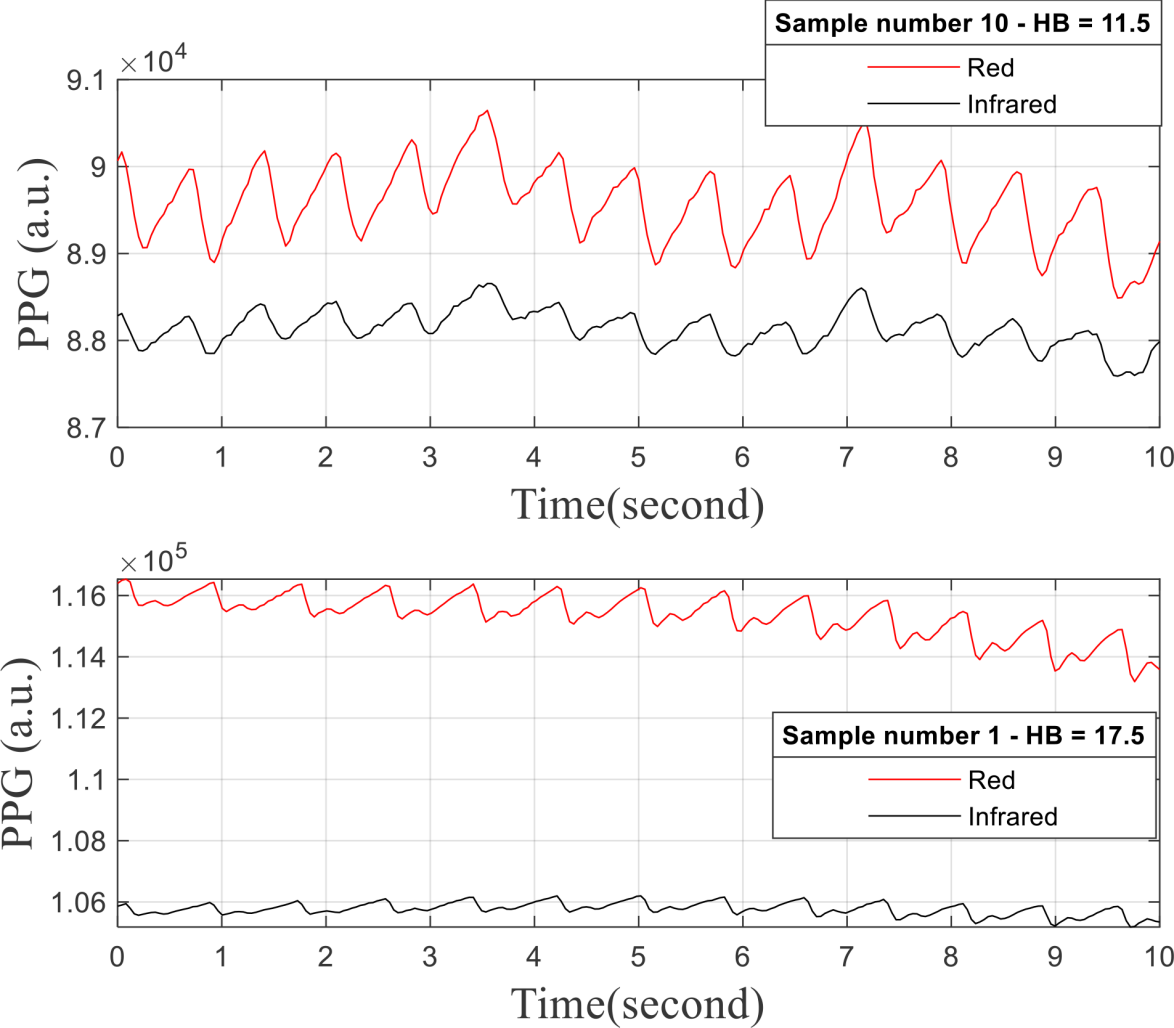
Comparative PPG signal traces for different hemoglobin levels. data plotted from Abuzairi et al (Abuzairi et al., 2024).

The data gathering technique, as detailed by Abuzairi et al (Abuzairi et al., 2024), involved the production of two different forms of light by the MAX30102 sensor: red light at 660 nm and infrared light at 880 nm. The skin’s light absorption was measured using a photodetector, and the data were then transmitted to an Arduino Uno via the I2C protocol for processing. The transformation of these raw signals into meaningful red and infrared light values was accomplished using the ‘MAX30105.h’ library, which employs the ‘getRawValues()’ method. Subsequently, the modified data were sent to a Python application using the ‘serial.Serial()’ method, where the baud rate and port settings were adjusted to align with those of the Arduino Uno. The PPG signals of each of the 68 participants were recorded for 10 seconds at 40 ms intervals, resulting in a total of 250 data sets. During the data collection, room temperature and illumination were maintained constant. Hemoglobin levels were simultaneously analyzed using a painful method involving a blood sample drawn via a finger prick, utilizing a Nesco Multicheck 2^®^ hemoglobinometer from Bioptik Technology. This blood sample, along with the light intensity data from the PPG signals and the hemoglobin levels measurements, was examined on-site to generate a complete dataset for further analysis. The changes in red and infrared light absorption over a 10-second period are depicted in The changes in red and infrared light absorption over a 10-second period are depicted in **Figure 2** (adapted from Abuzairi et al (Abuzairi et al., 2024)), showcasing the PPG signal traces for two separate samples. The light absorption properties captured by the PPG sensor are influenced by changes in hemoglobin levels, and these traces are essential for understanding this relationship. The top graph, showing Sample 10 with a hemoglobin levels of 11.5 g/dL, reveals a clear oscillation of the red signal above the infrared, while both red and infrared light signals are displayed. This pattern is indicative of how the optical qualities of the tissue at lower hemoglobin levels and the blood’s hemoglobin levels affect certain light absorption and attenuation characteristics. The bottom graph, displaying Sample 1 with a hemoglobin levels of 17.5 g/dL, attenuate the anticipated variations in light absorption with increasing hemoglobin levels, with the red light signal exhibiting stronger amplitude oscillations than the infrared. The flatter slope shown in the graph could be due to more consistent signal detection or altered physiological reactions resulting from elevated hemoglobin levels. The amplitude of the PPG signals, which are crucial for the subsequent analysis and feature extraction to non-invasively predict hemoglobin levels, is made readily visible on both graphs with differing y-axes scaling.

### 2.3 Feature extraction

The feature extraction process for the photoplethysmography (PPG) signals involved computing mean, kurtosis, and skewness from both red and infrared signals, selected for their ability to capture physiological characteristics relevant to hemoglobin prediction. The mean represents the average intensity of the PPG signal, providing a baseline measure of light attenuation influenced by blood volume and hemoglobin concentration. Kurtosis quantifies the peakedness of the signal waveform, indicating variations in blood volume pulsations that may reflect hemoglobin-related hemodynamic changes. Skewness measures the asymmetry of the signal distribution, capturing potential non-linear hemodynamic patterns correlated with hemoglobin levels.

Introduction to Key Features: Mean, Kurtosis, and Skewness.

1. Mean (μ): The mean of a signal represents the average value, providing a basic measure of the central tendency of the data points over time. In the PPG signals, as shown in Equation 1, the mean can indicate the overall light absorption level, which is influenced by the blood volume in the microvascular bed under the sensor.

(1) $$\mu =\frac{1}{N}\sum_{i=1}^{N}x_{i},$$

where x_i_​ is the i-th PPG signal sample, and N is the total number of samples in the signal.
2. Kurtosis (K): As shown in Equation 2, Kurtosis measures the “tailedness” of the probability distribution of a real-valued random variable. High kurtosis in a PPG signal can indicate more pronounced peaks, suggesting variability in blood volume during each cardiac cycle.

(2) $$K=\frac{\frac{1}{N}\sum_{i=1}^{N}{\left(x_{i}-\mu \right)}^{4}}{{\left(\frac{1}{N}\sum_{i=1}^{N}{\left(x_{i}-\mu \right)}^{2}\right)}^{2}},$$

where x_i_​ is the i-th PPG signal sample, μ is the mean of the signal, and N is the total number of samples.
3. Skewness (S): Skewness measures the asymmetry of the data around the sample mean. In the PPG signals, as shown in Equation 3, skewness can help identify asymmetries in the waveform, which may be indicative of physiological conditions affecting blood flow dynamics.

(3) $$S=\frac{\frac{1}{N}\sum_{i=1}^{N}{\left(x_{i}-\mu \right)}^{3}}{{\left(\frac{1}{N}\sum_{i=1}^{N}{\left(x_{i}-\mu \right)}^{2}\right)}^{3/2}},$$

where x_i_​ is the i-th PPG signal sample, μ is the mean, and N is the total number of samples.

For each subject in the dataset, these three features were extracted from both the red and infrared signals. The ability to capture these statistical properties from both light spectrums allows for a nuanced analysis of blood dynamics, as different wavelengths penetrate tissues differently. Alongside these six features (three from each spectrum), demographic data such as age and gender were also included, resulting in a total of eight features per sample.

The feature extraction process for the PPG signals involved the computation of mean, kurtosis, and skewness, selected for their ability to encapsulate distinct physiological and signal characteristics relevant to hemoglobin prediction. The mean represents the average intensity of the PPG signal, providing a baseline measure of light absorption influenced by blood volume and hemoglobin concentration. Kurtosis was chosen to quantify the peakedness or sharpness of the signal waveform, which is indicative of variations in blood volume pulsations and may reflect hemoglobin-related hemodynamic changes. Skewness, measuring the asymmetry of the signal distribution, was included to capture potential non-linear hemodynamic patterns that could correlate with hemoglobin levels.

### 2.4 Multilayer perceptron neural network

An MLP is a class of feedforward ANN that consists of at least three layers of nodes: an input layer, one or more hidden layers, and an output layer. Each node, or neuron, in one layer connects with a certain weight to every node in the following layer, making these networks well-suited for complex pattern recognition tasks such as classification and regression. In the context of classifying PPG signals, MLPs are particularly valuable due to their ability to learn non-linear models and their robustness in handling noise associated with PPG data. The multilayer structure allows MLPs to model complex relationships between the input features and the target variables. This capability is crucial when working with PPG signals, where the objective is to interpret subtle variations in light absorption that correlate with variations in blood volume and hemoglobin levels. The fundamental operation of an MLP involves forward propagation, as shown in Equations 4, 5, where inputs are passed through successive layers using weights and biases, and backpropagation, where errors are used to adjust those weights. Here’s a simplified view of the computations involved (Gallant and White, 1992; Taylor, 1996):

1. Forward Propagation:

(4) $$z^{\left(l\right)}=W^{\left(l\right)}a^{\left(l-1\right)}+b^{\left(l\right)}$$

(5) $$a^{\left(l\right)}=\text{σ}\left(z^{\left(l\right)}\right)$$

Here $z^{\left(l\right)}\;$is the input to layer l, and $b^{\left(l\right)}\;$are the weights and biases for layer l, $a^{\left(l\right)}$ is the activation at layer l, and σ represents the activation function.
2. Backpropagation: As shown in Equation 6, the network minimizes a loss function, typically mean squared error for regression tasks:

(6) $$L=\frac{1}{2N}\sum \;{\left(y_{true}-y_{pred}\right)}^{2}$$

Here, L is the loss, N is the number of samples, $y_{true}$ are the actual values, and $y_{pred}$ are the predicted values from the network.

For designing and testing the MLP structure, MATLAB software was utilized due to its comprehensive suite of tools for neural network training and evaluation. MATLAB’s Neural Network Toolbox provides a user-friendly interface for constructing various network architectures, training them with different algorithms, and testing their performance. To address concerns about overfitting and underfitting, given the relatively small sample size of 68 participants, the dataset was randomly split at the patient level into 70% for training and 30% for testing using stratified sampling based on age groups and sex, ensuring all PPG records from a given subject were assigned to either the training or testing set to prevent data leakage. This approach maintained proportional representation across age groups and sexes in both sets, enhancing the model’s generalizability. This balanced approach, combined with strong model performance, reassures that the model is not biased and can perform reliably across different population segments. Multiple network structures were tested, ranging from one hidden layer to four hidden layers, incorporating different numbers of neurons and activation functions to determine the optimal architecture. Each configuration was evaluated based on its ability to predict hemoglobin levels accurately. The validity of the model was ensured by selecting the most effective model based on its performance across both training and testing datasets, thereby minimizing risks of overfitting and bias. Various network configurations were tested, and the model that performed best on both datasets was chosen. The final model, which demonstrated high accuracy in predicting hemoglobin levels, showcases the effectiveness of MLPs in handling the complexity of PPG-derived data. In this study, the hyperparameters for the MLP model were selected through empirical testing, and the following setup was found to produce the best results. The **Table 1** summarizes the key hyperparameters and their rationale.

**Table 1:** Summary of hyperparameters used in the MLP model.

Hyperparameter	Value	Explanation
Number of Hidden Layers	2	After testing various architectures, two hidden layers provided the best performance without overfitting.
Number of Neurons (Hidden Layer 1)	20	The first hidden layer has 20 neurons, which provided the highest accuracy and allowed for sufficient complexity without overfitting.
Number of Neurons (Hidden Layer 2)	8	The second hidden layer was optimized with 8 neurons, which contributed to better performance while minimizing computational cost.
Epochs	1000	1000 epochs were used to ensure convergence, with early stopping applied to prevent overfitting.
Activation Function (Hidden Layers)	Tansig (Hyperbolic Tangent Sigmoid)	This activation function was chosen for its ability to capture non-linear relationships, essential for PPG signal processing.
Activation Function (Output Layer)	Linear	The linear function was used in the output layer to predict continuous values like hemoglobin concentration.
Learning Rate	0.01	A learning rate of 0.01 was selected based on preliminary experiments to ensure stable convergence.

## 3 Results

This research aimed to develop and validate a predictive model using an MLP neural network to predict hemoglobin levels from PPG signals non-invasively. The study utilized a dataset comprising both red and infrared PPG signal features, including mean, kurtosis, and skewness, alongside demographic variables such as age and gender. These variables were processed through feature extraction techniques to prepare them for neural network analysis. The model was trained to learn the underlying patterns correlating these features with hemoglobin levels, aiming to provide a reliable, non-invasive method for hemoglobin predicting. The MLP neural network designed for this study comprised eight input features and a single output. The input features included three extracted features each from the red and infrared PPG signals (mean, kurtosis, skewness), along with the subject’s age and gender. The sole output of the network was the predicted hemoglobin level.

**Figure 3:**
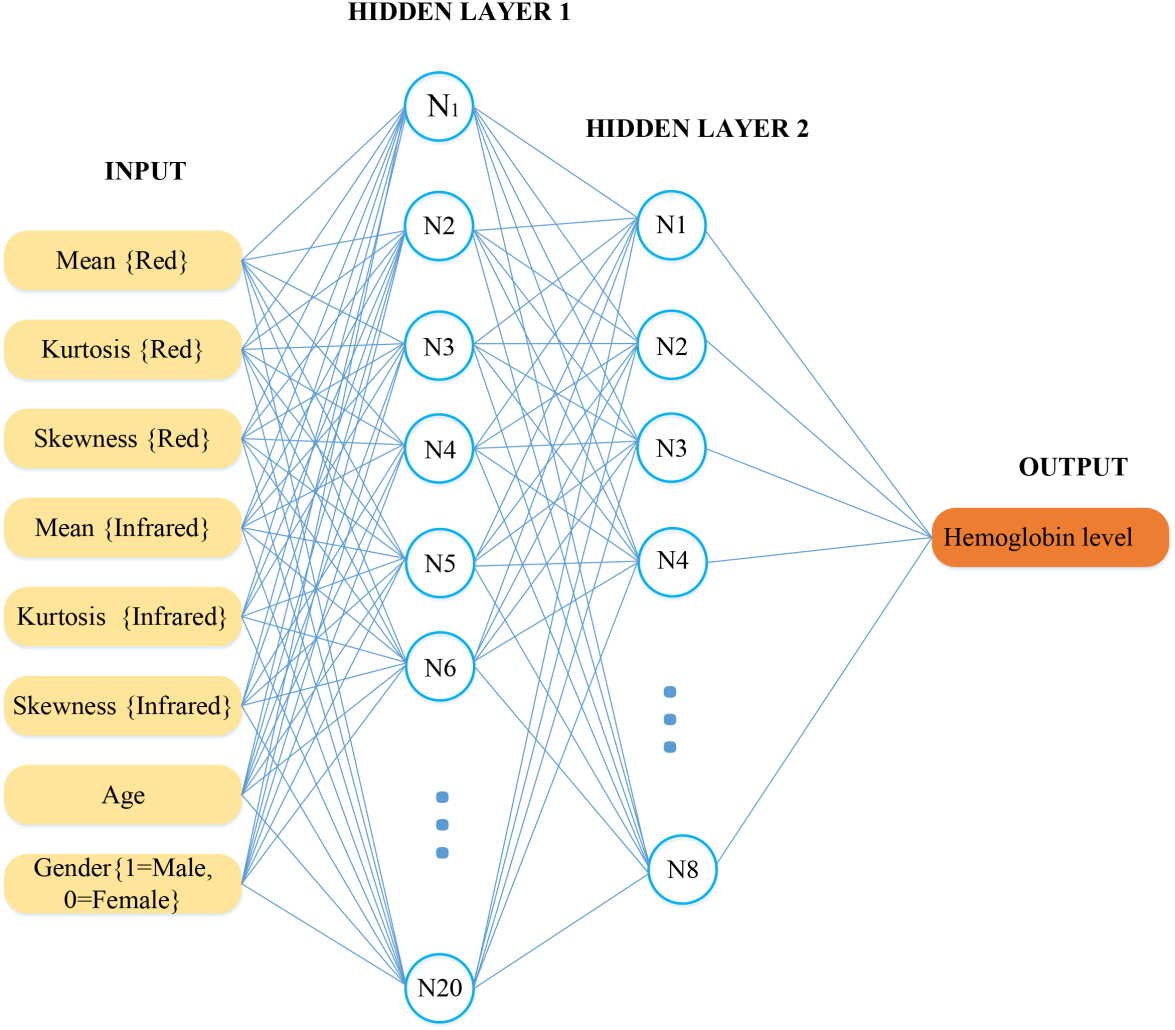
Neural network architecture for hemoglobin prediction.

The neural network architecture, as depicted in **Figure 3**, consists of an input layer with eight neurons representing each feature, two hidden layers, and an output layer that predicts the hemoglobin value. The activation function for the hidden layers was the hyperbolic tangent sigmoid function (tansig), which is effective for capturing nonlinearities in complex biological data. In contrast, the input and output layers used a linear activation function, suitable for continuous value prediction like hemoglobin levels.

**Figure 4:**
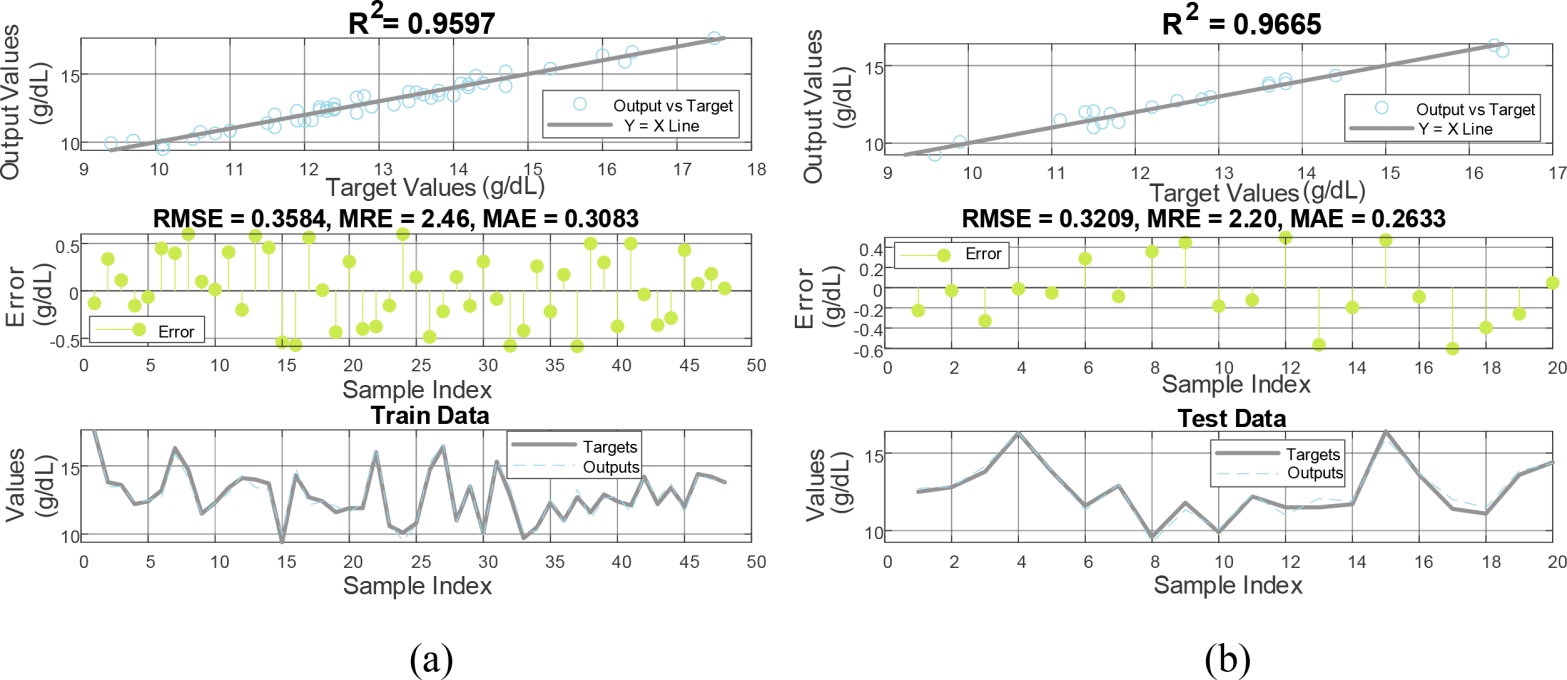
Evaluation of MLP model performance on **(a)** training and **(b)** testing datasets. This image presents regression, error, and fit plots, demonstrating strong correlation (R² > 0.95), low errors (MRE ~2.2-2.46%), and close matches between predicted and actual Hb levels.

**Figure 4** provides a detailed analysis of the performance of the designed MLP neural network in estimating hemoglobin levels across both the training and testing datasets. Each dataset is evaluated through three types of visual representations: regression analysis, error distribution, and fit plots. The regression plots for both datasets display a strong linear relationship between the predicted outputs and the target hemoglobin levels, with high correlation coefficients indicating robust predictive accuracy. Specifically, the model achieves an R² value of 0.9597 for the training dataset and 0.9665 for the testing dataset, confirming that the MLP model has successfully captured the underlying patterns in the data.

Error plots further validate the model’s performance, illustrating the residuals or deviations between the predicted and actual hemoglobin levels. In the training set, the residuals are mostly clustered close to zero with an RMSE of 0.3584 and an MRE of 2.4644, demonstrating minimal errors in predictions. Similarly, the testing set maintains a low error profile with an RMSE of 0.3209 and an MRE of 2.1997, emphasizing the model’s precision and consistency across unseen data. Additionally, the MAE for the training set is 0.3083 and for the testing set is 0.2633, further reinforcing the model’s accuracy.

Fit plots provide a direct comparison of the actual and predicted hemoglobin levels, showing close matches throughout the data sequence. This consistent accuracy across both training and testing phases highlights the MLP’s capability to generalize well beyond the data it was trained on, confirming its utility for real-world applications in non-invasive hemoglobin measurement.

The error metrics, including MAE, RMSE, and R² values, along with the MRE, demonstrate that the model is not only accurate but also capable of generalizing well to unseen data. The low RMSE and MRE values indicate minimal discrepancies between predicted and actual values, further validating the effectiveness of the MLP model. These results significantly enhance our understanding of non-invasive hemoglobin measurement using PPG signals. The high accuracy and low error rates demonstrate that MLP networks, equipped with well-selected features from PPG signals, can reliably predict hemoglobin levels. This supports the potential of PPG as a practical tool for routine hemoglobin monitoring, reducing the need for invasive blood tests and facilitating continuous health monitoring. The demonstrated high accuracy of the proposed MLP model in determining hemoglobin levels from PPG signals marks a significant advancement in non-invasive medical diagnostics. By leveraging deep learning techniques, this approach can be further refined and possibly integrated into wearable health monitoring devices, providing real-time, accurate hemoglobin measurements.

**Figure 5:**
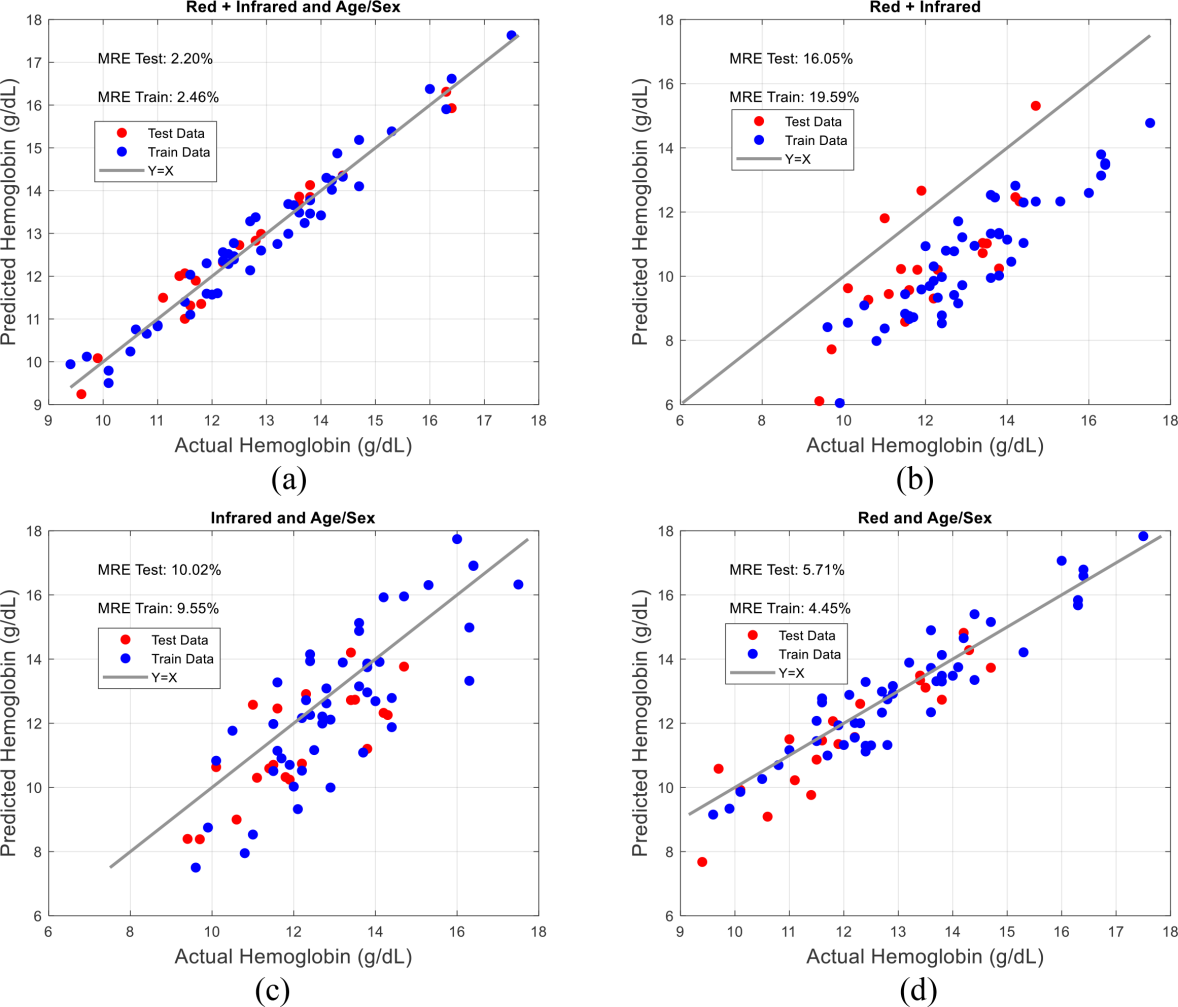
Ablation study results comparing the performance of models trained on different feature sets: **(a)** Red + infrared with age/sex, **(b)** Red + infrared without age/sex, **(c)** Red + age/sex, **(d)** Infrared + age/sex.

To evaluate the impact of different feature combinations, an ablation study was conducted in which separate models were trained using various subsets of features. Specifically, models were trained using only Red features (and the patient’s age/sex), only Infrared features (and the patient’s age/sex), and Red + Infrared features (with and without the inclusion of age/sex information). The comparison of these models, shown in **Figure 5**, highlights the contribution of each feature set to the overall model performance. The MRE for each model configuration is as follows: Red + Infrared with age/sex yielded MRE Test: 2.20%, MRE Train: 2.46% (**Figure 5a**); Red + Infrared without age/sex resulted in MRE Test: 16.05%, MRE Train: 19.59% (**Figure 5b**); Red + Age/Sex showed MRE Test: 10.02%, MRE Train: 9.55% (**Figure 5c**); and Infrared + Age/Sex achieved MRE Test: 5.71%, MRE Train: 4.45% (**Figure 5d**).

**Figure 6:**
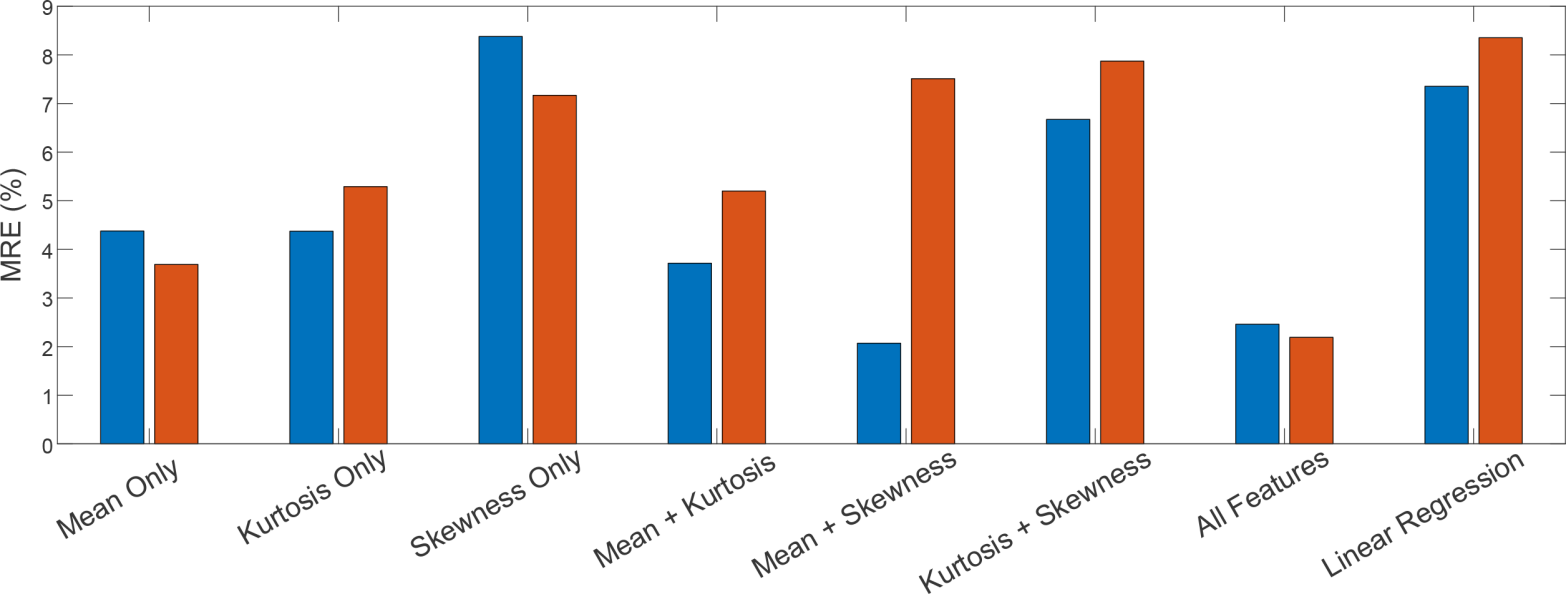
Comparison of MRE for models trained on different feature sets.

To evaluate the importance of each feature, the performance of the model was compared using different feature sets. The results, shown in **Figure 6**, compare the performance of models trained on individual features (mean, skewness, kurtosis), combinations of features (mean + kurtosis, mean + skewness, kurtosis + skewness), and the full set of features. Additionally, a simple linear regression model using only the mean was included as a baseline for comparison. The MRE values were calculated for both train and test datasets, demonstrating the relative effectiveness of the various configurations. The results indicate that the all features model performs the best, but a linear regression model using just the mean still performs reasonably well, confirming that the model benefits from the inclusion of additional features, but also that a simple approach could yield decent results.

**Figure 7:**
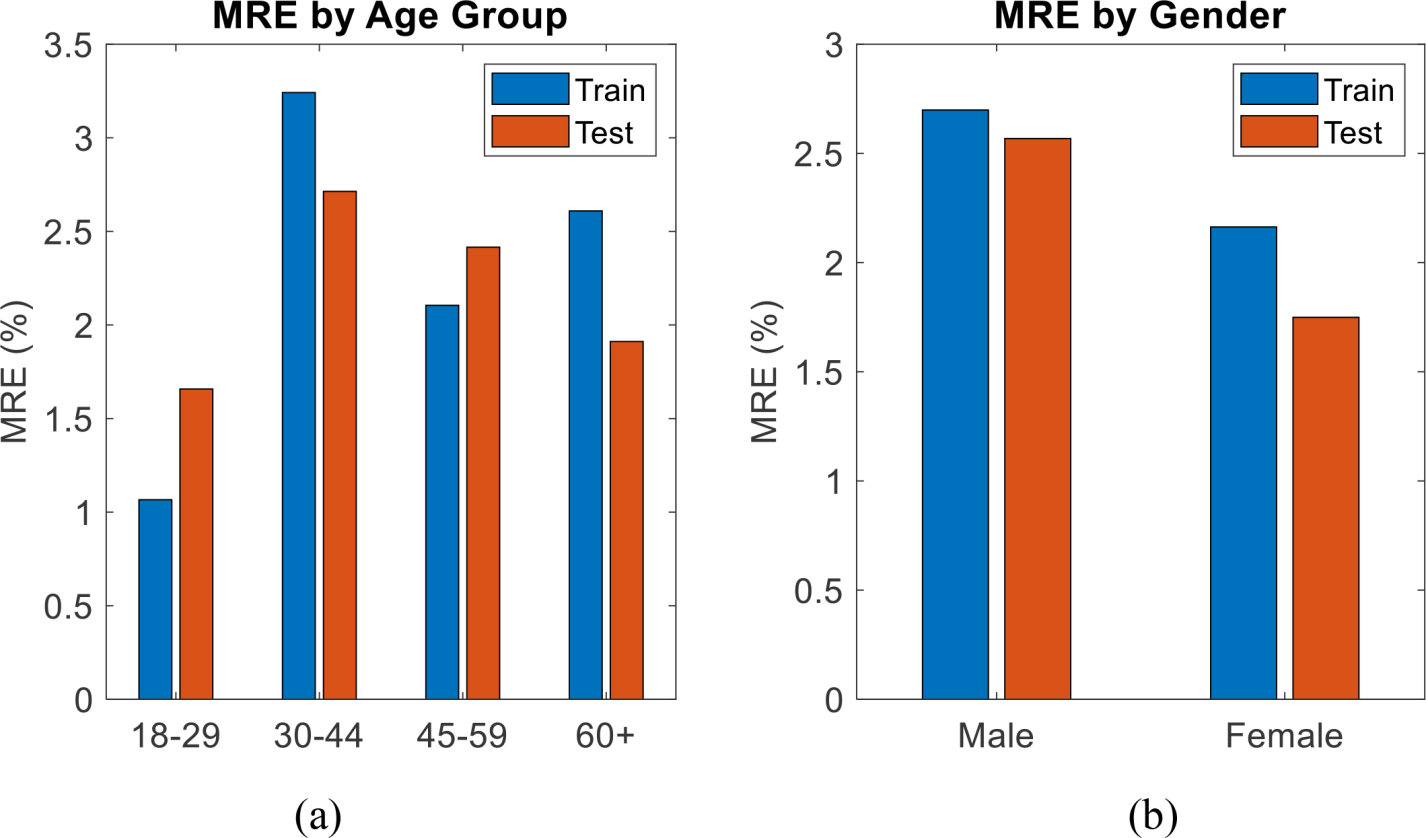
MRE performance of the model for test data segmented by **(a)** age group and **(b)** gender.

To further assess the effectiveness of the model across different demographics, the results have been expanded to include the MRE for the train and test data segmented by age ranges and sex. The analysis, shown in **Figure 7**, consists of two bar charts: one showing performance by age group (**Figure 7a**) and the other by gender (**Figure 7b**). The results highlight that the model performs consistently across different demographic groups, with only minor variations in MRE across age groups and sexes. The age group analysis reveals that the model performs best for the 30–44 age range, while the sex-based analysis shows slightly higher errors for males compared to females. These results further confirm the robustness of the proposed system across diverse patient populations.

To rigorously assess the robustness of the Multilayer Perceptron (MLP) model and minimize variability due to random data partitioning in our small dataset (n=68 subjects), we performed subject-level stratified 10-fold cross-validation repeated 100 times independently, each with a different random seed. This ensured balanced age and sex distributions across folds and complete subject separation to prevent data leakage. The validation procedure was inspired by the approach in Hackstein et al. (2021) (Hackstein et al., 2021), who employed 100 independent 10-fold cross-validations in a similarly small clinical cohort (n=55 subjects) to demonstrate the stability of their classification model despite limited data. The overall mean relative error (MRE) was 2.55%, calculated as the mean of 1, 000 individual MRE values derived from 100 independent repetitions of subject-level stratified 10-fold cross-validation (10 folds per repetition). The corresponding standard deviation (SD) of 1.05% was computed directly from these 1, 000 individual values. **Table 2** summarizes the mean MRE for groups of 10 repetitions each (averaging 100 individual MRE values per group) for illustrative purposes. Accordingly, the SD calculated from these 10 group-level means differs from the overall SD of 1.05%, as it reflects only between-group variability following within-group averaging.

**Table 2:** Mean relative error (MRE, in %) across 100 independent repetitions of subject-level stratified 10-fold cross-validation.

Group of Repetitions	Repetitions Included	Number of Folds per Repetition	Total evaluations per group	Mean MRE per group (%)
Group 1	1–10	10	100	1.50
Group 2	11–20	10	100	3.60
Group 3	21–30	10	100	1.80
Group 4	31–40	10	100	3.40
Group 5	41–50	10	100	2.20
Group 6	51–60	10	100	2.80
Group 7	61–70	10	100	1.70
Group 8	71–80	10	100	2.90
Group 9	81–90	10	100	3.30
Group 10	91–100	10	100	2.30

## 4 Discussion

In this study, the selected features—mean, kurtosis, and skewness—were chosen based on their effectiveness in capturing key characteristics of the PPG signal that are relevant to non-invasive hemoglobin predicting. These features are not only well-established in the field of signal processing but also computationally efficient, making them ideal for real-time applications with limited resources. The MRE of our model was 2.46%, which demonstrates that these features were highly appropriate for the task, as confirmed by the model’s performance on both training and testing datasets. **Table 3** presents a comprehensive comparison of our proposed MLP model against recent non-invasive hemoglobin estimation studies (Lakshmi and Manimegalai, 2019; Hasan et al., 2021; Yılmaz et al., 2022; Sabith et al., 2024), with metrics verified from original sources and standardized to three decimal places for equitable evaluation. Notably, while (Yılmaz et al., 2022) achieves a marginally lower MAPE (2.090%) using a computationally intensive combined deep learning framework on a large dataset (353 subjects), our model delivers superior RMSE (0.321 g/dL vs. 0.560 g/dL) with a shallower architecture and smaller cohort (68 subjects), underscoring its precision in absolute error terms. Similarly (Yılmaz et al., 2022),’s stacked regression benefits from synthetic data augmentation (MAPE = 6.000%, RMSE = 0.960 g/dL), yet lags in error reduction compared to our feature-extracted PPG approach. As a review (Hasan et al., 2021), highlights benchmarks like HemaApp’s RMSE (1.260 g/dL), which our method outperforms by ~75%, while (Lakshmi and Manimegalai, 2019)’s PCA-ANN (RMSE = 0.663 g/dL) demonstrates the value of dimensionality reduction—echoed in our statistical features—but without demographic integration. Overall, these comparisons affirm our model’s competitive accuracy, with added emphasis on its lightweight design for practical POC deployment, as elaborated in the following discussion on efficiency and regulatory compliance.

**Table 3:** Comparison of the proposed model’s performance with recent studies.

Study	Method	MRE/MAPE (%)	RMSE (g/dL)	R²
Proposed	MLP + Feature Extraction (Mean, Kurtosis, Skewness from Red/IR PPG) + Demographics	2.460 (MRE)	0.321	0.967
(Hasan et al., 2021)	Review of ANN/Smartphone-based Methods (e.g., HemaApp: MLR on RGB PPG)	N/A	1.260	0.820
(Lakshmi and Manimegalai, 2019)	PCA + ANN	N/A	0.663	0.770 (R)
(Yılmaz et al., 2022)	Combined Deep Learning (CNN on Nail Images + Numerical Features)	2.090 (MAPE)	0.560	N/A
(Sabith et al., 2024)	Improved Stacked Regression (XGBoost/Lasso + GAN-Augmented Data)	6.000 (MAPE)	0.960	>0.900

**Table 3** provides an illustrative comparison of performance metrics with selected recent non-invasive hemoglobin estimation studies (Lakshmi and Manimegalai, 2019; Hasan et al., 2021; Yılmaz et al., 2022; Sabith et al., 2024). Direct comparisons are limited by substantial differences in dataset size, demographic diversity, PPG hardware, reference standards, and methodological approaches. Notably, while some studies report marginally lower MAPE on larger cohorts, our lightweight MLP architecture achieves competitive RMSE (0.321 g/dL) and demonstrates promising internal precision in a considerably smaller dataset (n=68 subjects), highlighting the potential efficiency of targeted statistical feature extraction and demographic augmentation for resource-constrained applications.

The competitive accuracy of our MLP model, as evidenced in **Table 3**, extends to its practical deployment advantages, particularly in offline processing, which is essential for resource-limited clinical environments. Unlike cloud-dependent deep learning approaches in (Yılmaz et al., 2022), which incur inference latencies of seconds to minutes due to data transmission and server processing, our model completes predictions in 0.1–0.5 seconds on edge devices such as Arduino microcontrollers integrated with the MAX30102 sensor. This offline capability eliminates reliance on stable internet connectivity, making it viable for remote or underserved settings where network disruptions are common. Furthermore, local computation safeguards patient privacy by avoiding the transmission of sensitive health data (e.g., PPG signals and hemoglobin estimates), aligning with stringent regulations. The European Union’s General Data Protection Regulation (GDPR) Article 9 prohibits processing special categories of personal data, including health metrics, without explicit consent, and explicitly restricts bulk collection on remote servers to prevent re-identification risks. Similarly, the U.S. Health Insurance Portability and Accountability Act (HIPAA) Security Rule (45 CFR § 164.312) requires safeguards against unauthorized transmission of protected health information, limiting unencrypted cloud uploads to mitigate breach risks. Violations can lead to substantial penalties, including fines up to €20 million (4% of global turnover) under GDPR or $50, 000 per incident under HIPAA. By confining all operations to the device, our method ensures compliance, accelerates regulatory pathways (e.g., FDA Class II clearance), and reduces hardware costs by 50–70% through integration into low-power wearables, thereby enhancing scalability for anemia screening and chronic monitoring in diverse populations. To demonstrate the sufficiency of the MLP—a relatively simple feedforward architecture—for hemoglobin prediction, we compared its performance against advanced models in recent publications (Lakshmi and Manimegalai, 2019; Hasan et al., 2021; Yılmaz et al., 2022; Sabith et al., 2024), as detailed in **Table 3**. Despite the modest dataset size (n=68 subjects, a noted limitation), the proposed model yields performance metrics that are comparable to or better than those reported in prior studies. This outcome can be attributed to the deliberate selection of features capturing statistical properties (mean, kurtosis, skewness) from dual-wavelength PPG signals, combined with demographic variables (age and gender). Ablation analyses substantiate that integration of demographic factors (age and gender) enhances performance stability, resulting in an approximately 14% reduction in Mean Relative Error (MRE). Potential overfitting was effectively mitigated through rigorous subject-level stratified 10-fold cross-validation repeated 100 times independently.

While inference times for our MLP and more complex deep learning models (e.g (Yılmaz et al., 2022)) are comparable (both<0.5 seconds on standard hardware), the simplicity of the MLP extends beyond training efficiency to critical deployment advantages in resource-constrained environments. With approximately 500–1000 floating-point operations (FLOPs) per prediction—versus 10^6–^10^8^ FLOPs for deep CNNs—our model requires minimal memory (~10–50 KB parameters) and computational power, enabling seamless integration into low-cost edge devices like the Arduino Uno paired with MAX30102 sensor (total processing: 2–3 seconds, including feature extraction). This contrasts with resource-intensive models that often necessitate cloud offloading, introducing latency, connectivity dependencies, and privacy risks under regulations like GDPR Article 9 and HIPAA Security Rule. By supporting fully offline, real-time hemoglobin monitoring in wearables or point-of-care settings, our approach enhances accessibility for underserved populations, reducing costs by 50-70% compared to hardware for advanced models.

These results demonstrate that the model performs best when both Red and Infrared features are used in combination with age and sex information, as seen in **Figure 5a**. The inclusion of age and sex significantly improves the model’s accuracy, as evidenced by the comparison between **Figures 5b, d**, where the exclusion of these demographic features causes a noticeable increase in the MRE. This emphasizes the importance of demographic data in enhancing model performance. Additionally, the Red + Infrared without age/sex configuration in **Figure 5b** showed the highest error, indicating that the absence of these features compromises the model’s ability to predict hemoglobin levels accurately.

The results obtained from the MLP model in predicting hemoglobin levels using PPG signals provide significant insights into the field of non-invasive medical diagnostics. The high R^2^-values exceeding 0.96 for both training and testing datasets underscore the model’s robustness and predictive accuracy. This high level of accuracy indicates that the selected features—mean, kurtosis, skewness, age, and gender—adequately capture the essential dynamics of the PPG signals related to hemoglobin levels. The model’s ability to closely predict hemoglobin levels from these signals suggests that PPG, when coupled with advanced machine learning techniques, can serve as a reliable tool for monitoring vital blood parameters without the need for invasive procedures. This advancement has the potential to revolutionize patient care, particularly in settings where traditional blood sampling is impractical or undesirable. Moreover, the successful application of MLP highlights the potential of deep learning in enhancing the diagnostic processes by providing fast, accurate, and non-invasive alternatives. These findings contribute to the broader knowledge base by demonstrating the feasibility of using neural networks to interpret complex biological signals, potentially paving the way for their application in other areas of health monitoring.

While this study demonstrates promising results, several limitations warrant consideration. The dataset’s modest size (n=68 subjects) and limited diversity (adult-only, balanced sex but narrow age range and health status) constrain generalizability across broader demographics and physiological variations, including skin tones or comorbidities; future work should incorporate larger, heterogeneous cohorts for enhanced validation. Additionally, the model’s reliance on the MAX30102 sensor within a 3D-printed fingertip shell and Arduino setup may hinder adaptability to alternative PPG hardware, while the design’s partial susceptibility to ambient light interference—despite efforts to minimize it—could degrade signal quality in variable lighting conditions, as advanced systems like Masimo’s SpHb employ superior shielding. The neural network architecture, optimized for this dataset, requires testing on divergent data characteristics to confirm robustness. Reference hemoglobin measurements via a single finger-prick with the Nesco Multicheck 2^®^ hemoglobinometer, a portable but non-gold-standard tool with potential accuracy limitations relative to laboratory analyzers, may introduce reference variability; moreover, the absence of Bland-Altman analysis limits insights into method agreement and biases. To mitigate these, subsequent studies should adopt multiple measurements or lab-grade analyzers for references, integrate Bland-Altman plots, and explore adaptive signal processing or diverse hardware. Notably, the system’s total processing time (12–13 seconds: 10 seconds for PPG acquisition + 2–3 seconds for feature extraction and prediction) supports real-time applicability, though optimization could further streamline it for clinical use. Another important limitation of this study is the lack of investigation into the effects of common comorbidities on PPG-based hemoglobin estimation. Conditions such as hypertension, diabetes mellitus, cardiovascular diseases, and chronic kidney disease are highly prevalent in clinical populations and may influence PPG signal characteristics through alterations in vascular compliance, peripheral perfusion, and microcirculatory function. These pathophysiological changes could potentially affect the accuracy of our model. Future studies should include patients with various underlying health conditions to evaluate the robustness and generalizability of the proposed method across diverse clinical scenarios. Additionally, stratified analyses based on comorbidity profiles would help identify any condition-specific calibration requirements or performance variations.

The accuracy and reliability of our non-invasive hemoglobin prediction model may be influenced by confounding factors that affect PPG signal quality, particularly light attenuation by non-hemoglobin-containing media such as melanin and water. Melanin, present in varying concentrations across different skin tones, absorbs light in the red (660 nm) and infrared (880 nm) spectra, potentially altering PPG signal characteristics and introducing variability in predictions. Similarly, differences in tissue water content among subjects, which also exhibits light attenuation properties in these wavelengths, may contribute to inter-subject variability in PPG signals. Other factors, such as skin thickness or subcutaneous fat, could further impact signal quality. These confounding factors represent a limitation of our study, as they were not explicitly accounted for in the model. Future studies should incorporate advanced signal processing techniques, such as adaptive filtering or normalization for skin tone and tissue composition, to mitigate these effects and improve the model’s robustness across diverse populations. The results of this study have significant practical implications, particularly in low-resource and clinical settings. The non-invasive nature of the hemoglobin prediction method, combined with the use of affordable PPG sensors, could provide an accessible solution for monitoring hemoglobin levels in regions with limited access to traditional laboratory tests. This method could be integrated into wearable devices or mobile applications, allowing for continuous, real-time monitoring of patients, particularly in remote or underserved areas. However, there are several limitations to consider when applying this method in real-world scenarios. One key challenge is the potential impact of skin tone variations on PPG signal quality. Skin pigmentation can influence the absorption and scattering of light, potentially leading to variations in the signal that might affect the accuracy of hemoglobin predicting. Additionally, motion artifacts in the PPG signal, such as those caused by physical movement or poor sensor placement, could lead to inaccurate readings. To mitigate these challenges, future research could explore advanced signal processing techniques or the integration of additional sensors to improve robustness in diverse real-world environments.

Future research should prioritize expanding the dataset to encompass larger, more diverse cohorts, including varied age groups, ethnic backgrounds, and health conditions, to evaluate model robustness and generalizability. Testing the framework across alternative PPG sensors and hardware configurations will clarify adaptability and support clinical standardization. Incorporating additional PPG-derived features (e.g., entropy or frequency-domain metrics) alongside other physiological inputs could further refine predictive accuracy. The selected features—mean, kurtosis, and skewness—effectively summarize PPG signal characteristics (intensity, distribution, asymmetry), as evidenced by the model’s strong performance (R² > 0.96) on both training and testing sets, justifying their suitability for hemoglobin estimation. While these suffice for the current task, future explorations of advanced neural architectures or machine learning algorithms may yield comparative insights and enhanced handling of PPG variability. The present study provides a preliminary demonstration of the feasibility of non-invasive hemoglobin estimation using multi-wavelength PPG signals and a lightweight MLP model in a small clinical cohort. Although extensive repeated stratified cross-validation (100 independent repetitions of 10-fold CV, totaling 1000 evaluations) indicates promising internal stability (MRE 2.55% ± 1.05% SD), the study remains limited by reliance on a single, relatively small dataset (n=68 subjects) acquired at one clinical center. Consequently, broader generalizability across diverse populations, devices, skin tones, comorbidities, and clinical environments cannot yet be established, and definitive proof-of-concept status would require external validation. As with other preliminary PPG-based investigations, comprehensive confirmation of performance will necessitate substantially larger, ideally multi-center datasets, including out-of-distribution cohorts.

## 5 Conclusion

This study provides a preliminary demonstration of the feasibility of non-invasive hemoglobin estimation using multi-wavelength PPG signals and a lightweight MLP neural network in a small clinical cohort of 68 subjects. Extensive internal validation through subject-level stratified 10-fold cross-validation repeated 100 times independently yielded a Mean Relative Error (MRE) of 2.55% ± 1.05% SD. In contrast to many existing PPG-based approaches that employ computationally intensive deep learning architectures or require large-scale data augmentation, the present framework utilizes lightweight statistical features (mean, kurtosis, skewness) extracted from dual-wavelength signals, augmented by demographic variables (age and gender). Ablation analyses confirm that demographic integration substantially enhances performance, reducing MRE by approximately 14%. The resulting model demands minimal computational resources (~500–1000 FLOPs per prediction), facilitating offline execution on low-cost edge devices such as Arduino-integrated MAX30102 sensors. This design supports compliance with privacy regulations (e.g., GDPR Article 9, HIPAA Security Rule) by eliminating the need for cloud-based processing and provides reduced latency and deployment costs (estimated 50–70% lower than cloud-dependent alternatives). The approach thus offers potential for rapid, patient-friendly hemoglobin monitoring without invasive procedures, with applications in anemia screening, transfusion guidance, and chronic disease management, particularly in resource-constrained or remote settings. However, given the reliance on a single, relatively small dataset acquired at one clinical center, broader generalizability across diverse populations, devices, skin tones, comorbidities, and clinical environments remains to be established through larger, ideally multi-center external validation studies. The current findings lay a preliminary foundation for scalable AI-driven diagnostics and encourage future investigations incorporating more diverse cohorts and multi-parameter sensing modalities.

## Data Availability

The original contributions presented in the study are included in the article/**Supplementary Material**. Further inquiries can be directed to the corresponding author.
